# Physician and Surgeon

**Published:** 1900-11-17

**Authors:** 


"PHYSICIAN AND SURGEON."

The title and description by which the London L.S.A. is in future to be known is "Physician and Surgeon." As we lately stated the Society of Apothecaries undertook some time ago to protect any of its licentiates from the consequences of assuming the title of "Physician and Surgeon," and they have now resolved that this is the only title which the Society can authorise as a proper description of those holding the qualification L.S.A., 1886. This may either be added to title of L.S.A. or used alone The Society has also decided to discourage, in every possible way, the use of any other titles by their licentiates. This is as it should be. The L.S.A. is a good, old, and respectable qualification, and in view of the fact that, while the old title of apothecary has practically lapsed and become meaningless, the L.S.A. is now a fully qualified practitioner of both medicine and surgery, it seems but right that he should be given a title which shall express his position. But the claim to have all sorts of fancy letters after his name, which has been put forward by some writers, is absurd, and should be at once put down.

